# Penile Prosthesis Implantation in a Patient With Post-polio Residual Paralysis and Pelvic Deformity: A Case Report

**DOI:** 10.7759/cureus.115377

**Published:** 2026-08-28

**Authors:** Praveen B Joshi, Sai Dilip Sriramula

**Affiliations:** 1 Department of Urology and Andrology, Joshi's Andrology and Urology Centre, Bangalore, IND

**Keywords:** erectile dysfunction, malleable implant, pelvic deformity, penile prosthesis, poliomyelitis

## Abstract

Penile prosthesis implantation is the gold-standard treatment for men with erectile dysfunction refractory to conservative therapies. Surgical implantation may become technically challenging in patients with significant musculoskeletal deformities because altered pelvic anatomy can affect patient positioning and corporal access. We report the case of a 50-year-old man with type 2 diabetes mellitus and post-polio residual paralysis associated with fixed pelvic deformity who presented with severe erectile dysfunction refractory to phosphodiesterase type 5 inhibitors, low-intensity extracorporeal shockwave therapy, and intracavernosal injections. A Shah malleable penile prosthesis was successfully implanted through a penoscrotal approach despite difficulties related to pelvic tilt and altered corporal orientation. Careful patient positioning, meticulous corporal dilatation, and intraoperative modification of the surgical technique enabled successful implantation without complications. The postoperative course was uneventful, and at four weeks, the patient demonstrated satisfactory wound healing without evidence of infection, erosion, or device-related complications and reported satisfaction with the early surgical outcome. This case highlights the technical considerations required during penile prosthesis implantation in patients with post-polio musculoskeletal deformities and emphasizes that successful outcomes can be achieved with appropriate preoperative planning, careful surgical technique, and individualized perioperative management.

## Introduction

Erectile dysfunction affects a substantial proportion of middle-aged and older men, with prevalence increasing with advancing age and the presence of comorbid conditions such as diabetes mellitus, cardiovascular disease, and neurological disorders. Although penile prosthesis implantation remains the definitive treatment for refractory erectile dysfunction, coexisting musculoskeletal deformities may introduce unique technical challenges related to patient positioning, pelvic anatomy, and corporal access that are infrequently discussed in the literature [1,2].

Post-polio residual paralysis (PPRP) is characterized by asymmetric lower limb wasting, joint contractures, and pelvic tilt resulting from anterior horn cell damage during acute poliomyelitis [3-5]. Although India is now a polio-free nation, a significant cohort of survivors with PPRP continues to age and develop urological problems including erectile dysfunction. Published literature describing penile prosthesis implantation in patients with PPRP and fixed pelvic deformity appears to be extremely limited. We report this case to highlight the technical challenges encountered during surgery and to discuss practical operative considerations that may assist surgeons managing patients with similar musculoskeletal deformities.

## Case presentation

A 50-year-old man presented with a 10-year history of severe erectile dysfunction. His medical history was significant for type 2 diabetes mellitus (T2DM) of 18 years' duration, with reasonable glycemic control at the time of surgery (HbA1c 7.2%). He had a history of poliomyelitis in childhood, resulting in right upper limb weakness, right lower limb wasting, and a fixed pelvic tilt secondary to PPRP. He had failed sequential treatment with oral phosphodiesterase type 5 inhibitors, low-intensity extracorporeal shockwave therapy, and intracavernosal injections. His International Index of Erectile Function (IIEF) score [6] was 7, consistent with severe erectile dysfunction. The preoperative stretched penile length (SPL) was 11 cm.

The preoperative assessment included evaluation of pelvic tilt and hip range of motion to anticipate potential challenges with patient positioning and surgical access. Pelvic radiography was performed to assess the underlying pelvic asymmetry.

The patient was positioned in a modified supine position with additional padding beneath the hip, and the right lower limb was used as a support to accommodate the pelvic asymmetry. A vertical penoscrotal approach was used.

Intraoperatively, the right corpus cavernosum was found to be tilted and rotated anteriorly, resulting in the posterior displacement of the left corpus cavernosum. Figure 1 provides a diagrammatic representation of the cross-sectional anatomy of the penis, with Figure 1A demonstrating the normal anatomical relationship between the urethra and both corpora cavernosa and Figure 1B illustrating the altered anatomy in this patient, characterized by posterior tilting of the left corpus cavernosum and deviation of the urethra from its normal position.

**Figure 1:**
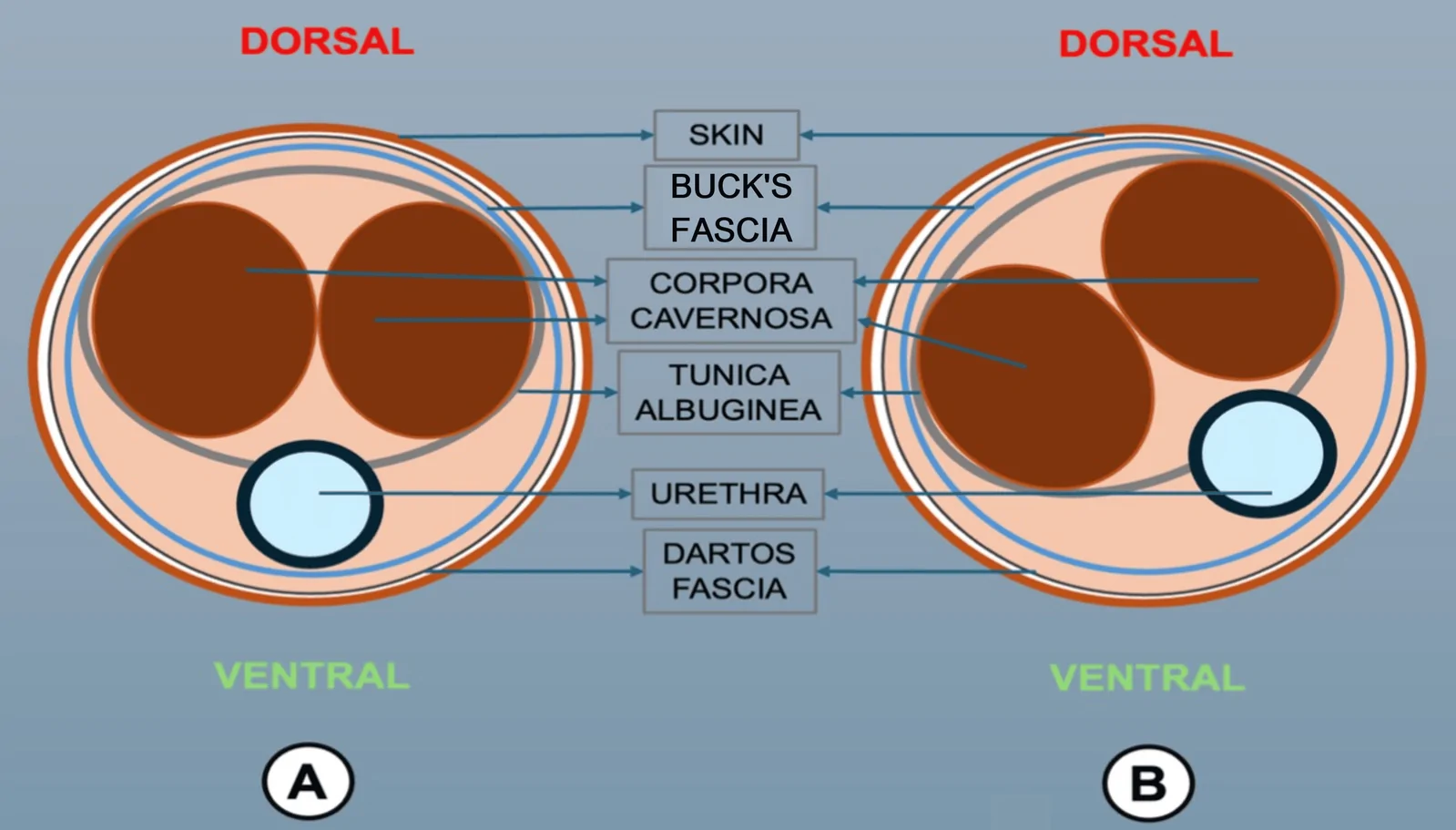
Schematic representation of corporal rotation in post-polio pelvic deformity Illustration depicting the altered orientation of the corporal bodies due to poliomyelitis. The right corpus cavernosum is rotated anteriorly, while the left corpus cavernosum lies relatively posteriorly, highlighting the anatomical challenges encountered during penile prosthesis implantation. The figure was created manually by the authors using Microsoft PowerPoint (Microsoft Corporation, Redmond, Washington, United States) based on the intraoperative findings.

Manual detorsion of the penile shaft was performed during placement of the stay sutures, corporotomy, and corporal dilatation to bring the left corpus cavernosum into a more anterior position. The urethra was carefully retracted away from the operative field to minimize the risk of urethral injury. These additional surgical maneuvers, together with appropriate modification of patient positioning, facilitated safe corporal dilatation and prosthesis placement without intraoperative complications.

A Shah malleable penile prosthesis (WH-11) was successfully implanted. Intraoperative corporal measurements demonstrated a 7-mm discrepancy in corporal length, measuring 19.3 cm on the right and 20 cm on the left. Following the removal of the outer sleeves of the cylinders, the prostheses were successfully inserted into both corpora, with the distal tips extending to the mid-glans. The total operative time was 150 minutes.

Postoperatively, the patient experienced mild discomfort during mobilization, attributed to his underlying musculoskeletal imbalance and the recent surgical intervention. He was discharged on postoperative day 2. At the four-week postoperative follow-up, wound healing was satisfactory, with no evidence of surgical site infection, prosthesis erosion, or other complications (Figure 2). At eight weeks postoperatively, the patient was able to engage in satisfactory sexual intercourse.

**Figure 2:**
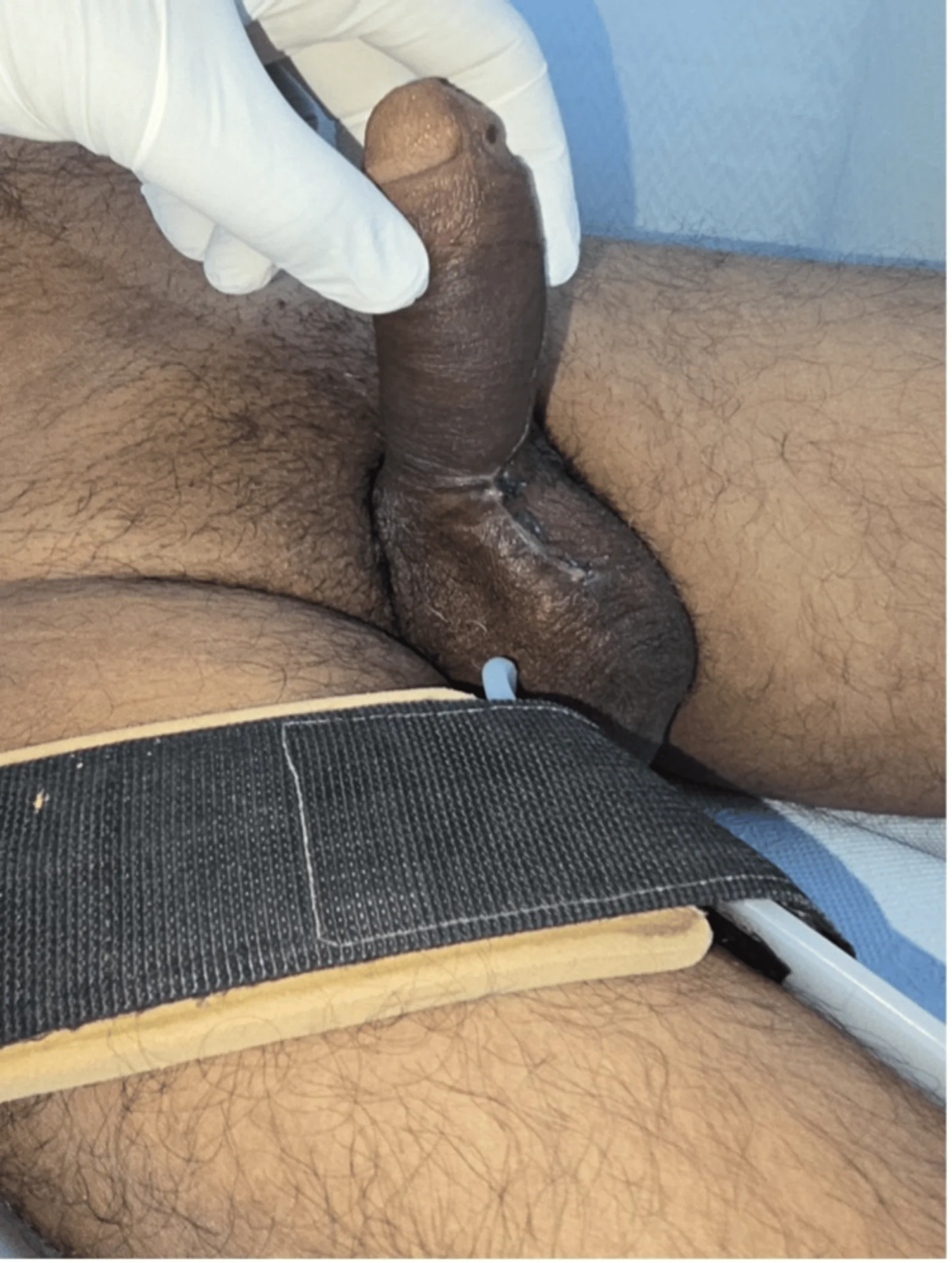
Four-week postoperative appearance following Shah malleable penile prosthesis implantation Clinical photograph obtained four weeks after surgery demonstrating satisfactory wound healing following the implantation of a Shah malleable penile prosthesis through a vertical penoscrotal approach.

## Discussion

Reports describing penile prosthesis implantation in patients with PPRP and fixed pelvic deformity are exceedingly limited. The present case illustrates several unique operative challenges related to altered pelvic alignment and corporal orientation that have received little attention in the available literature.

Diabetes mellitus independently increases the risk of penile prosthesis implantation-related infection and corporal fibrosis [7]. Our patient had well-controlled HbA1c at the time of surgery, and adherence to established perioperative infection-prevention protocols, as outlined in our institute's antibiotic protocol, mitigated the risk of infection [8-10]. Good glycemic control, appropriate prosthesis selection, and adherence to infection-prevention protocols further contributed to favorable postoperative results. The choice of a malleable device was chosen by the patient over inflatable prostheses due to financial constraints.

Key lessons from this case include the following: (1) thorough preoperative assessment of pelvic tilt and hip mobility is essential for anticipating positioning and dilatation challenges; (2) additional padding and asymmetric positioning aids may be required intraoperatively; (3) careful appreciation of the altered penile anatomy is crucial, to prevent misidentification of the urethra for the corpus cavernosum; and (4) postoperative mobilization planning should account for the underlying musculoskeletal condition [2,11,12]. Multidisciplinary input from physiotherapy and orthopedics may be beneficial in select patients.

This case adds to the limited literature on penile prosthesis implantation in patients with significant musculoskeletal deformities and may provide practical guidance for urologists managing similar patients in the future.

## Conclusions

Penile prosthesis implantation in patients with PPRP and associated pelvic deformity was technically feasible in our patient despite the unique anatomical challenges posed by pelvic tilt and musculoskeletal asymmetry. This case demonstrates that careful preoperative assessment, meticulous patient positioning, recognition of altered corporal anatomy, and individualized modification of the surgical technique can facilitate successful implantation in selected patients with similar anatomical challenges. Further clinical experience is needed to determine the generalizability of these observations.
